# The effectiveness of frequent antibiotic use in reducing the risk of infection-related hospital admissions: results from two large population-based cohorts

**DOI:** 10.1186/s12916-020-1504-5

**Published:** 2020-03-02

**Authors:** Tjeerd Pieter van Staa, Victoria Palin, Yan Li, William Welfare, Timothy W. Felton, Paul Dark, Darren M. Ashcroft

**Affiliations:** 1 Centre for Health Informatics, Division of Informatics, Imaging and Data Science, Faculty of Biology, Medicine and Health, School of Health Sciences, The University of Manchester, Manchester Academic Health Science Centre, Vaughan House, Manchester, M13 9PL UK; 2 Utrecht Institute for Pharmaceutical Sciences, Utrecht University, Utrecht, the Netherlands; 3 Public Health England North West, 3 Piccadilly Place, London Road, Manchester, M1 3BN UK; 4 Division of Infection, Immunity and Respiratory Medicine, Faculty of Biology, Medicine and Health, The University of Manchester, Manchester Academic Health Science Centre, Manchester, UK; 5 Intensive Care Unit, Manchester University NHS Foundation Trust, Wythenshawe Hospital, Manchester, UK; 6 Centre for Pharmacoepidemiology and Drug Safety, NIHR Greater Manchester Patient Safety Translational Research Centre, School of Health Sciences, Faculty of Biology, Medicine and Health, The University of Manchester, Oxford Road, Manchester, M13 9PL UK

**Keywords:** Antibiotics, Primary care, Effectiveness, Infection-related complications, Epidemiology

## Abstract

**Background:**

Previous research reported that individuals prescribed antibiotics frequently develop antimicrobial resistance. The objective of this study was to evaluate whether frequent antibiotic use is associated with reduced hospital admissions for infection-related complications.

**Methods:**

Population-based cohort study analysing electronic health records from primary care linked to hospital admission records. The study population included patients prescribed a systemic antibiotic, recent record of selected infections and no history of chronic obstructive pulmonary disease. Propensity-matched cohorts were identified based on quintiles of prior antibiotic use in 3 years before.

**Results:**

A total of 1.8 million patients were included. Repeated antibiotic use was frequent. The highest rates of hospital admissions for infection-related complications were observed shortly after antibiotic start in all prior exposure quintiles. For patients with limited prior antibiotic use, rates then dropped quickly and substantially. In contrast, reductions over time were substantially less in patients with frequent prior antibiotic use, with rates remaining elevated over the following 6 months. In patients without comorbidity comparing the highest to lowest prior exposure quintiles in the Clinical Practice Research Databank, the IRRs were 1.18 [95% CI 0.90–1.55] in the first 3 days after prescription, 1.44 [95% CI 1.14–1.81] in the days 4–30 after and 3.22 [95% CI 2.29–4.53] in the 3–6 months after.

**Conclusions:**

Repeated courses of antibiotics, although common practice, may have limited benefit and indicator of adverse outcomes. A potential mechanism is that antibiotics may cause dysbiosis (perturbations of intestinal microbiota), contributing to colonization with resistant bacteria. Antibiotics should be used judiciously and only periodically unless indicated. Antimicrobial stewardship should include activities focusing on the substantive number of patients who repeatedly but intermittently get antibiotics.

## Introduction

Antimicrobial resistance (AMR) is a major public health concern resulting in increased morbidity, mortality and healthcare costs. Without additional action to tackle AMR, many common healthcare interventions and procedures could become too risky to undertake. Internationally, an estimated 700,000 deaths are attributed to AMR annually [1]. In the UK, primary care accounts for 81% of antibiotic prescribing in England [2]. Many initiatives have been taken to reduce the levels of antibiotic use by clinicians. In primary care in England, these include the development and implementation of the TARGET toolkit, feedback to prescribers and the recent Quality Premium focusing on antibiotic use for urinary tract infections (UTIs) [3, 4].

The optimal level of antibiotic use or exposure characteristics (such as duration and repeated use over time) associated with the best clinical outcomes are not known. A study of common respiratory tract infections found that lower levels of antibiotic use were associated with a higher incidence of pneumonia and peritonsillar abscess [5]. A systematic literature review showed that individuals prescribed an antibiotic for a respiratory or urinary infection in primary care are more likely to develop a resistant infection in the following months. The effect was greatest in the month immediately after treatment [6]. However, any long-term persistence and clinical implications of AMR were not addressed in this review. A large longitudinal analysis of urine cultures found that resistance to an antibiotic is strongly related to prior antibiotic use [7]. Repeat prescribing of antibiotics is widespread in UK primary care [8], but there is limited evidence whether this is a clinically effective and safe strategy. The overall aim of this study was to evaluate whether frequent antibiotic use is associated with reduced hospital admissions for infection-related complications.

## Methods

### Database

This study used data from two sources which included the Clinical Practice Research Databank (CPRD) and the Secure Anonymized Information Linkage (SAIL) databases. CPRD contains longitudinal, anonymized, patient-level electronic health records (EHRs) from general practices in the UK with more than five million active patient records representing about 8% of the UK population [9]. SAIL contains data from general practices in Wales covering 75% of the population in Wales (about three million people) [10, 11]. The EHRs include the clinical diagnoses, medication prescribed, vaccination history, diagnostic testing, lifestyle information, clinical referrals, and patient’s age, sex, ethnicity, smoking history and body mass index (BMI). The patient-level data from the general practices has been linked through a trusted third party to hospital admission data (hospital episode statistics) using unique patient identifiers [9]. The hospital data contained information on the date of hospital admission and the clinical diagnoses established at and during admission and coded using ICD10. Linked data were available for about half of CPRD practices which are all located in England and for all the SAIL practices. Only data from the linked CPRD practices were used. Patient-level socioeconomic information was available through linkage of the postcode of a patient’s residence to the Index of Multiple Deprivation (IMD) [12]. Patient-level IMD was aggregated into quintiles for the current analysis. Prescriptions were classified using the British National Formulary (BNF) sections. The rationale for using both CPRD and SAIL was to replicate results in two independent population-based cohorts of patients from England and Wales. CPRD was used to develop the analytical strategy followed by replication in SAIL. This replication approach was used as the research hypothesis was not defined a priori*.* The CPRD and SAIL databases include broadly comparable study populations (although from different nations in the UK) using a similar healthcare system. Although there were many similarities between the two databases, different coding systems (versions of the Read coding dictionary), EHR software systems and data preparation procedures were used in CPRD and SAIL.

### Study population

The study population consisted of patients who were prescribed a systemic antibiotic in their general practice (BNF chapter 5.1, except 5.19, 5.1.10, 5.1.11 including oral, rectal or intravenous applications; topical applications were excluded). The CPRD study population included patients prescribed an antibiotic between January 2000 and June 2015; for SAIL, this time was between January 2000 and December 2017 (the end dates represented the date of the latest data collection). Analyses included all antibiotic prescriptions recorded in the EHR follow-up with a diagnosis of a common infection such as upper respiratory tract infection (URTI), lower respiratory tract infection (LRTI), UTI and otitis media and externa at the index date or month before. Patients with COPD or multiple common infections were excluded. To evaluate the long-term effects of antibiotics, the study population was restricted to patients with at least 3 years of prior history in the patient EHR (i.e. time between the prescription and start of EHR follow-up for a patient). The exposure of interest was the number of antibiotic prescriptions in the 3 years before divided into quintiles (five groups). Patients could move over time between different quintiles as prior history was evaluated at each individual antibiotic prescription (although patients could only belong to a single quintile at a given point in time). Four pairs of propensity-matched cohorts were then randomly selected in order to compare the higher quintiles to the quintile with the lowest history of antibiotic use. Propensity scores are the probability of assignment to one exposure conditional on a patient’s measured baseline covariates. Patients in the different quintiles of histories of prior antibiotic were matched by these propensity scores, initially by the rounded propensity score (two decimals) followed by matching within the caliper (i.e. pre-specified maximum difference). The caliper was taken as 0.20 of the logit of the propensity score as recommended in the literature [13].

**Fig. 1:**
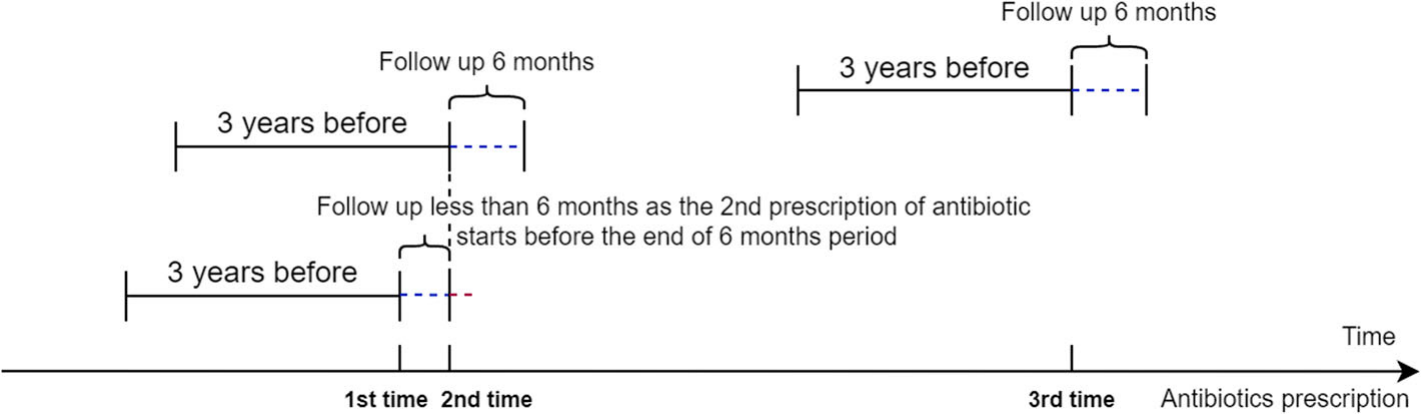
Diagram of the design of exposure measurement (history of antibiotic prescribing in 3 years before) and follow-up for infection-related hospitalizations (in the 6 months after)

The primary outcome of interest was hospital admission with infection-related complication (as recorded in HES) that occurred in the 30 or 182 days after the antibiotic prescription. Follow-up was reset to zero in case of a repeat antibiotic prescription in the follow-up period. The hospital admissions for infection-related complications were based on the primary admission diagnosis with the broad set of infections (such as hospital admission for LRTI, pneumonia, sepsis). The predefined ICD10 codes included A37-A41, A46, A48, A49, B95, B96, G00, G01, G04-G08, H60, H65-H68, H70, H71, I00-I02, I33, I38, I39, J00-J06, J13-J18, J20-J22, J36, J40-J42, J85, J86, K05, K35- K37, K61, K81, L00, L01-L05, L08, M00, M01, M86, N10, N30, N45, N70-N75, O23, O85, O86, O91, O98, P23, P36, and P37. Code lists used in this study are available on the Clinical Codes repository [14]. Figure 1 provides a diagrammatic representation of the design of exposure measurement and follow-up for the outcomes of interest.

Four different analyses were conducted in this study. The first analysis compared the risk of clinical outcomes between patients with different levels of prior antibiotic prescriptions in the 3 years before. The risks of clinical outcomes were compared between quintiles with different histories of antibiotic use stratified by different time periods after the antibiotic prescription. The first 3 days after were considered, pragmatically, to reflect the severity of the underlying infection while more distant time was considered to reflect the treatment effects of antibiotics and that of the changes in infection severity. Incidence rate ratios (IRRs) were estimated for different periods comparing the quintiles of history of prior antibiotic use. The second analysis visualized the incidence rates of infection-related hospital admissions for each quintile of prior antibiotic use. IRRs were estimated for each time unit (day or week). The IRRs were then smoothed (due to instability of the individual estimates) [15]. A series of sensitivity analyses was also conducted. One evaluated the clinical outcomes in all antibiotic prescriptions (i.e. those with any or no recorded indication) and others evaluated the risks stratified by age and calendar time. Also, analyses were also conducted evaluating individual common infections. Another sensitivity analyses considered the clinical codes that were recorded on the date of the antibiotic prescriptions or in the 6 months before. These codes were extracted and compared between the patients in the lowest and highest quintiles of prior antibiotic use (restricted to codes that were recorded > 50 times). This analysis was restricted to patients in the tertile of propensity scores that were most likely to have limited history of prior antibiotic use. The odds ratios (ORs) between the different history quintiles were estimated and ranked, followed by a review of the codes with an OR of 1.5 or more. The random selection of a single antibiotic prescription per person was another sensitivity analysis (in order to evaluate the correlation of effects within a patient). The final sensitivity analysis concerned the fitting of Cox proportional hazards model with a single record for each patient. Follow-up started at the date of the first antibiotic prescription and ended at the date of censoring, with exposures (time in the first 6 months after the prescription) fitted as time-dependent variables and with separate predictor for the time more than 6 months after an antibiotic prescription. The analyses were adjusted for the age, sex and risk factors at the start of follow-up (first antibiotic prescription).

### Statistical analysis

The propensity scores for assignment to the quintile with the lowest prior antibiotic use were estimated comparing each of the four quintiles with higher histories of antibiotic use compared to the lowest quintile. Logistic regression was used to estimate the propensity scores for each patient. The risk factors included in the logistic models were age, sex, calendar year and season, ethnicity, socioeconomic class (IMD), comorbidity based on the Charlson Comorbidity Score (composite score of history of selected chronic conditions [16]), record of flu vaccination in the year before, number of non-antibiotic prescriptions in the year before, and hospital referral and inpatient hospitalization in the year before. Negative binomial regression models were fitted to estimate incidence rate ratios (IRRs) and 95% confidence intervals (CIs) comparing the rates of infection-related hospital admissions after an antibiotic prescription in the four higher quintiles of history of prior antibiotic use to the lowest quintile. Logistic regression was used in the comparisons of clinical codes between the highest and lowest quintiles of prior antibiotic use. The false discovery rate-adjusted *P* values were estimated in order to minimize the effects of multiple testing and the finding of false-positive statistical associations. All analyses were performed using SAS software version 9.4.

## Results

The source population of patients with over 3 years of follow-up included 2.1 million patients from CPRD and 0.6 million from SAIL. Totals of 11.5 million antibiotic prescriptions in CPRD and 3.7 million in SAIL were provided to these populations. In CPRD, 5.1 million antibiotic prescriptions had a recent record of one of the selected infections (URTI, LRTI, UTI, otitis media and externa), 2.0 million had a record of another infection and 4.3 million (37.8%) had no recorded indication. Table 1 shows the types of antibiotics prescribed at the index date stratified by the quintiles of prior antibiotic use. Amoxicillin (including co-amoxiclav) was the most frequently prescribed antibiotic although its use decreased in patients with higher prior antibiotic use. Use of clarithromycin, nitrofurantoin and cephalexin increased with higher prior antibiotic use. In both databases, many patients had a history of substantive prior use of antibiotics. Patients had received on average 7.1 antibiotic prescriptions in the 3 years before in CPRD and 6.6 in SAIL. 56.9% of antibiotic prescriptions (in CPRD) were prescribed to patients with 3+ prescriptions in the 3 years before and 18.9% to those with 9+ prior prescriptions. The prior number of antibiotics ranged in the lowest quintile from 0 to 1 in CPRD (0–1 in SAIL), low quintile 2 in CPRD (2–3 in SAIL), middle quintile 3–4 in CPRD (4–5 in SAIL), high quintile 5–8 in CPRD (6–9 in SAIL) and highest quintile 9+ in CPRD (10+ in SAIL).

**Table 1:** Counts (> 1%) of antibiotic types prescribed with recent record of selected infections stratified by quintile of prior antibiotic use (CPRD) (multiple types of antibiotics prescribed on the same date were counted separately)

	Quintile of prior antibiotic use				
	Lowest	Low	Middle	High	Highest
Amoxicillin	814,864 (52.5%)	321,935 (47.6%)	440,600 (45.0%)	416,928 (41.8%)	351,691 (35.1%)
Phenoxymethylpenicillin	232,338 (15.0%)	85,338 (12.6%)	107,876 (11.0%)	82,831 (8.3%)	40,654 (4.1%)
Trimethoprim	151,562 (9.8%)	68,977 (10.2%)	103,894 (10.6%)	110,187 (11.1%)	116,739 (11.6%)
Erythromycin	117,147 (7.6%)	58,666 (8.7%)	87,729 (9.0%)	88,363 (8.9%)	69,914 (7.0%)
Clarithromycin	62,377 (4.0%)	36,836 (5.4%)	59,371 (6.1%)	69,151 (6.9%)	75,576 (7.5%)
Cefalexin	51,594 (3.3%)	30,730 (4.5%)	51,907 (5.3%)	64,302 (6.5%)	90,273 (9.0%)
Doxycycline	36,885 (2.4%)	20,944 (3.1%)	34,639 (3.5%)	41,802 (4.2%)	55,612 (5.5%)
Nitrofurantoin	24,476 (1.6%)	16,431 (2.4%)	30,105 (3.1%)	41,386 (4.2%)	68,578 (6.8%)
Flucloxacillin	22,738 (1.5%)	11,063 (1.6%)	16,581 (1.7%)	17,847 (1.8%)	18,730 (1.9%)
Ciprofloxacin	14,853 (1.0%)	11,451 (1.7%)	21,166 (2.2%)	29,869 (3.0%)	56,727 (5.7%)
Cefaclor	7662 (0.5%)	5000 (0.7%)	8372 (0.9%)	10,583 (1.1%)	13,531 (1.3%)

The incidence of infection-related hospital admissions was 2.3 in CPRD and 6.1 in SAIL per 1000 person-months in the 30 days after (a total of 10,546 cases in CPRD and 7384 in SAIL). The incidence in children < 5 years was 1.8 in CPRD and 9.0 in SAIL, in patients aged 20–29 1.2 in CPRD and 3.7 in SAIL and in patients aged 80+ 8.4 in CPRD and 17.3 in SAIL. In CPRD, 48.0% of the infection-related hospital admissions were for LRTI, 33.9% for pneumonia and 10.1% for sore throat. In SAIL, these percentages were 29.2%, 36.4% and 26.7%, respectively.

Table 2 shows the characteristics of the propensity-matched cohorts of antibiotic users matching each of the different quintiles of prior antibiotic use to the lowest quintile. The four pairs of propensity-matched were well matched on age, sex and Charlson Comorbidity Score. The mean age in the pair with the lowest and low prior antibiotic use was 43 years, and the percentage of patients without comorbidity was 64%. This compares to a mean age of 50 years and 47% without comorbidity in the pair matching the highest to lowest quintile of prior antibiotic use.

**Table 2:** Characteristics of the propensity-matched cohorts of antibiotic users matching each of the different quintiles of prior antibiotic use to the lowest quintile

	Matched quintile of prior antibiotic use							
	Reference (lowest)	Low	Reference (lowest)	Middle	Reference (lowest)	High	Reference (lowest)	Highest
CPRD								
Total *N* antibiotic prescriptions	623,627	623,627	825,352	825,352	690,334	690,334	438,474	438,474
Age (mean)	43.4	43.3	44.3	44.2	45.9	45.7	50.9	50.7
Women (%)	398,289 (63.9%)	397,113 (63.7%)	548,995 (66.5%)	547,199 (66.3%)	477,977 (69.2%)	476,765 (69.1%)	315,692 (72%)	315,586 (72%)
Comorbidity score (%)								
Lowest (score 0)	399,315 (64.0%)	399,407 (64.0%)	503,555 (61.0%)	503,782 (61.0%)	390,999 (56.6%)	392,619 (56.9%)	207,908 (47.4%)	209,284 (47.7%)
Low (scores 1–2)	180,224 (28.9%)	179,917 (28.9%)	255,728 (31.0%)	255,218 (30.9%)	232,981 (33.7%)	231,525 (33.5%)	170,068 (38.8%)	169,353 (38.6%)
Middle (scores 3–4)	33,581 (5.4%)	33,681 (5.4%)	50,506 (6.1%)	50,647 (6.1%)	50,596 (7.3%)	50,269 (7.3%)	45,536 (10.4%)	45,106 (10.3%)
High (scores 5–6)	7633 (1.2%)	7697 (1.2%)	11,400 (1.4%)	11,515 (1.4%)	11,583 (1.7%)	11,696 (1.7%)	11,042 (2.5%)	10,856 (2.5%)
Highest (score 7+)	2874 (0.5%)	2925 (0.5%)	4163 (0.5%)	4190 (0.5%)	4175 (0.6%)	4225 (0.6%)	3920 (0.9%)	3875 (0.9%)
SAIL								
*N* antibiotic prescriptions (total)	246,887	246,887	159,261	159,261	141,620	141,620	102,947	102,947
Age (mean)	36.1	36.0	36.9	36.6	38.6	38.3	43.8	43.4
Women (%)	149,856 (60.7%)	149,799 (60.7%)	102,248 (64.2%)	101,854 (64%)	92,545 (65.3%)	92,484 (65.3%)	68,444 (66.5%)	68,816 (66.8%)
Comorbidity score (%)								
Lowest	202,665 (82.1%)	202,679 (82.1%)	123,976 (77.8%)	124,428 (78.1%)	105,998 (74.8%)	106,549 (75.2%)	70,005 (68.0%)	70,564 (68.5%)
Low	33,020 (13.4%)	33,000 (13.4%)	25,466 (16.0%)	25,193 (15.8%)	25,397 (17.9%)	25,004 (17.7%)	22,815 (22.2%)	22,407 (21.8%)
Middle	8473 (3.4%)	8493 (3.4%)	7339 (4.6%)	7177 (4.5%)	7660 (5.4%)	7498 (5.3%)	7591 (7.4%)	7490 (7.3%)
High	2005 (0.8%)	2015 (0.8%)	1819 (1.1%)	1804 (1.1%)	1887 (1.3%)	1914 (1.4%)	1881 (1.8%)	1850 (1.8%)
Highest	724 (0.3%)	700 (0.3%)	661 (0.4%)	659 (0.4%)	678 (0.5%)	655 (0.5%)	655 (0.6%)	636 (0.6%)

**Fig. 2:**
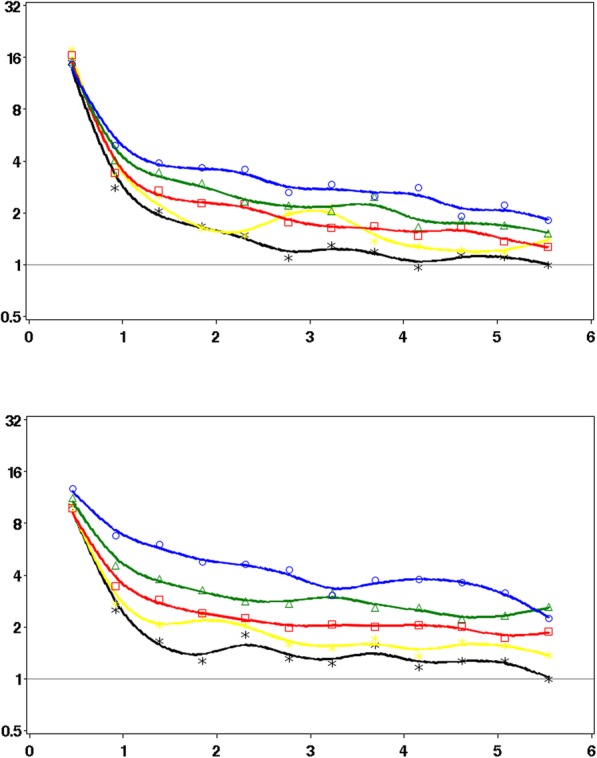
IRRs of hospital admissions for infection-related complications in the 6 months after stratified by quintile of prior antibiotic use (CPRD and SAIL)

**Fig. 3:**
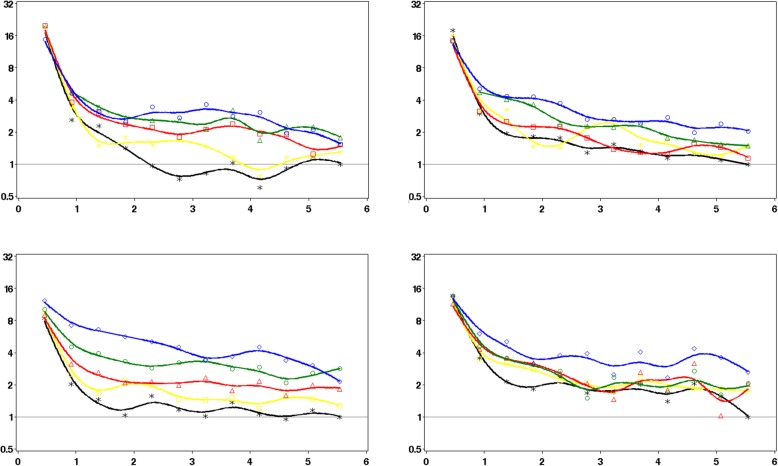
IRRs of hospital admissions for infection-related complications in the 6 months after stratified by the absence/presence of comorbidity (Charlson Comorbidity Score 0/1+) and quintile of prior antibiotic use (CPRD and SAIL). Asterisk indicates lowest (black), square indicates low (yellow), triangle indicates intermediate (red), diamond indicates high (green), circle indicates highest (blue); reference is month 6 in the lowest quintile. *Y* axis: IRRs; *X* axis: months after the antibiotic prescription

Figure 2 shows the patterns in the propensity-matched cohorts of the rates of infection-related hospital admissions in the 182 days after an antibiotic prescription stratified by prior history of antibiotic use. The highest rates were observed shortly after the antibiotic prescription in all groups. For patients with limited prior antibiotic use, the rates then dropped substantially. In contrast, the reductions over time were substantially less in patients with frequent prior antibiotic use, with rates remaining elevated. Results were broadly comparable between CPRD and SAIL. As shown in Fig. 3, stratification by comorbidity shows that the patterns of the rates of infection-related hospital admissions in the 182 days after an antibiotic prescription were broadly comparable between patients with and without comorbidity.

The adjusted IRRs of infection-related hospital admissions varied across the three different time periods after the antibiotic prescription and levels of prior antibiotic use (Table 3). In the first 3 days after an antibiotic prescription, the rates of infection-related hospital admission in CPRD were broadly similar between the groups (adjusted IRR of 0.90 [95% CI 0.77–1.04] in the highest compared to the lowest quintile of prior use). In the days 4–30 after, the rates were considerably higher with more frequent prior use (adjusted IRR of 1.52 [95% CI 1.34–1.72]). Differences between groups were even larger in the 3–6 months after (adjusted IRR of 2.26 [95% CI 1.92–2.67]). The results were broadly similar in SAIL. Comparable patterns of relative differences in the changes of risk of infection-related hospital admissions were found in patients with and without comorbidity.

**Table 3:** IRRs of infection-related hospital admissions in different time periods after an antibiotic prescription comparing different levels of prior antibiotic use (propensity-matched cohorts with URTI, LRTI, UTI, Otitis Media or Externa)

		CPRD			SAIL		
		Days 1–3 after	Days 4–30 after	Months 3–6 after	Days 1–3 after	Days 4–30 after	Months 3–6 after
Quintile of prior antibiotic use	Stratum	IRR (95% CI)	IRR (95% CI)	IRR (95% CI)	IRR (95% CI)	IRR (95% CI)	IRR (95% CI)
	All						
Lowest		Reference	Reference	Reference	Reference	Reference	Reference
Low		1.01 (0.88–1.16)	1.20 (1.05–1.37)	1.23 (1.07–1.42)	1.10 (0.96–1.25)	1.07 (0.95–1.19)	1.33 (1.20–1.48)
Middle		0.95 (0.84–1.07)	1.15 (1.03–1.28)	1.37 (1.21–1.55)	0.93 (0.79–1.09)	1.17 (1.03–1.33)	1.62 (1.43–1.82)
High		0.90 (0.79–1.02)	1.32 (1.18–1.49)	1.77 (1.57–2.00)	1.06 (0.91–1.25)	1.42 (1.25–1.61)	2.10 (1.87–2.36)
Highest		0.90 (0.77–1.04)	1.52 (1.34–1.72)	2.26 (1.92–2.67)	0.90 (0.74–1.08)	1.88 (1.63–2.16)	2.48 (1.17–2.84)
	No comorbidity^#^						
Lowest		Reference	Reference	Reference	Reference	Reference	Reference
Low		1.15 (0.93–1.41)	1.22 (1.00–1.49)	1.32 (1.04–1.66)	1.17 (1.00–1.36)	1.10 (0.96–1.26)	1.40 (1.23–1.58)
Middle		1.01 (0.84–1.22)	1.10 (0.93–1.31)	1.66 (1.34–2.07)	0.98 (0.81–1.19)	1.23 (1.04–1.45)	1.75 (1.51–2.03)
High		0.97 (0.79–1.20)	1.31 (1.08–1.59)	2.33 (1.88–2.87)	1.19 (0.97–1.47)	1.62 (1.37–1.91)	2.64 (2.29–3.02)
Highest		1.18 (0.90–1.55)	1.44 (1.14–1.81)	3.22 (2.29–4.53)	1.01 (0.79–1.30)	2.46 (2.00–3.02)	2.90 (2.44–3.45)
	Comorbidity^#^						
Lowest		Reference	Reference	Reference	Reference	Reference	Reference
Low		0.91 (0.75–1.10)	1.18 (0.99–1.41)	1.20 (1.00–1.43)	0.95 (0.74–1.21)	1.01 (0.84–1.22)	1.20 (0.99–1.44)
Middle		0.90 (0.77–1.05)	1.18 (1.02–1.36)	1.23 (1.05–1.43)	0.84 (0.64–1.11)	1.09 (0.89–1.34)	1.39 (1.13–1.70)
High		0.86 (0.73–1.01)	1.34 (1.16–1.55)	1.57 (1.33–1.84)	0.89 (0.69–1.16)	1.16 (0.95–1.43)	1.25 (1.01–1.54)
Highest		0.80 (0.67–0.95)	1.57 (1.35–1.82)	2.05 (1.69–2.47)	0.77 (0.58–1.02)	1.37 (1.12–1.67)	2.02 (1.58–2.58)

^#^Based on Charlson Comorbidity Score with score of 0 classified as no comorbidity and scores 1+ as comorbidity present

Table 4 shows the IRRs of infection-related hospital admissions stratified by the level of propensity score (i.e. likelihood of having limited history of prior antibiotic use). Patients most likely to have a limited history of prior antibiotic use were found to have the higher IRRs comparing the two higher quintiles to the lowest quintile. The IRR in CPRD was 5.72 (95% CI 2.08–15.76) with highest history of antibiotic use in the propensity tertile most likely to have limited history and 2.17 (95% CI 1.79–2.64) in the propensity tertile least likely to have limited history.

**Table 4:** IRRs of infection-related hospital admissions in months 3–6 after stratified by the level of propensity and quintile of prior antibiotic use

	Propensity for lowest prior antibiotic use					
	Low	Middle	High	Low	Middle	High
	% in groups^#^	% in groups^#^	% in groups^#^	IRR (95% CI)^&^	IRR (95% CI)^&^	IRR (95% CI)^&^
Quintiles of prior antibiotic use						
CPRD						
Low vs lowest	41.4% vs 29.4%	33.5% vs 32.8%	25.1% vs 37.8%	1.21 (1.02–1.45)	1.34 (1.00–1.79)	1.15 (0.75–1.75)
Middle vs lowest	45.8% vs 25.4%	33.4% vs 32.7%	20.8% vs 41.9%	1.38 (1.18–1.61)	1.38 (1.09–1.75)	1.33 (0.91–1.94)
High vs lowest	53.7% vs 21.3%	32.5% vs 33.3%	13.8% vs 45.4%	1.75 (1.48–2.06)	1.74 (1.38–2.19)	2.52 (1.45–4.37)
Highest vs lowest	69.1% vs 16.6%	25.7% vs 36.3%	5.2% vs 47.1%	2.17 (1.79–2.64)	2.88 (2.04–4.07)	5.72 (2.08–15.76)
SAIL						
Low vs lowest	41.7% vs 27.7%	33.5% vs 32.6%	24.8% vs 39.7%	1.22 (1.06–1.41)	1.42 (1.19–1.69)	1.56 (1.18–2.07)
Middle vs lowest	50.5% vs 25.3%	32.3% vs 33.4%	17.2% vs 41.4%	1.50 (1.29–1.74)	1.83 (1.44–2.34)	1.82 (1.28–2.59)
High vs lowest	57.5% vs 21.5%	30.5% vs 34.3%	12.0% vs 44.2%	1.72 (1.47–2.02)	2.62 (2.12–3.24)	3.67 (2.51–5.35)
Highest vs lowest	69.3% vs 15.8%	25.2% vs 37.1%	5.6% vs 47.2%	2.13 (1.74–2.61)	3.27 (2.60–4.12)	3.01 (1.69–5.37)

^#^Unmatched cohorts

^&^Propensity-matched cohorts

The sensitivity analyses of the IRRs of infection-related hospital admissions in months 3–6 after an antibiotic prescription are shown in Table 5. The effects were found to be largest in children and smallest in elderly. In the analysis of different calendar time periods, increased IRRs were found in more recent time (the mean number of prior antibiotics prescribed in the 3 years before was 5.8 during 2000–2004, 6.6 during 2005–2009 and 7.9 during 2010+ in CPRD). In the sensitivity analysis fitting a time-dependent Cox proportional hazards models with a single record per person, it was found that the adjusted IRRs were higher with increased quintiles of history of antibiotic use. In CPRD, the IRRs (listed by increasing quintile) were 1.32 (95% CI 1.23–1.42), 1.41 (95% CI 1.32–1.51), 1.73 (95% CI 1.62–1.84) and 2.18 (95% CI 2.03–2.33). In SAIL, these were 1.42 (95% CI 1.34–1.51), 1.75 (95% CI 1.64–1.88), 2.36 (95% CI 2.22–2.52) and 3.15 (95% CI 2.95–3.38).

**Table 5:** Sensitivity analyses of the IRRs of infection-related hospital admissions in month 3–6 after an antibiotic prescription (CPRD and SAIL)

		Quintile of prior antibiotic use				
			CPRD		SAIL	
		Lowest	High	Highest	High	Highest
Subgroup	Cohorts		Adjusted IRR (95% CI)	Adjusted IRR (95% CI)	Adjusted IRR (95% CI)	Adjusted IRR (95% CI)
Age < 18 years	Propensity-matched (URTI, LRTI, UTI, otitis media or externa)	Reference	3.10 (2.24–4.29)	5.14 (3.02–8.75)	3.16 (2.65–3.76)	3.69 (2.83–4.82)
Age 18–59 years		Reference	2.11 (1.61–2.75)	2.53 (1.74–3.67)	1.88 (1.46–2.42)	2.43 (1.73–3.41)
Age 60+ years		Reference	1.52 (1.29–1.79)	2.07 (1.74–2.48)	1.29 (1.06–1.58)	1.63 (1.28–2.07)
Calendar year 2000–2004		Reference	1.51 (1.05–2.17)	2.12 (1.33–3.40)	1.36 (0.95–1.95)	1.64 (1.07–2.52)
Calendar year 2005–2009		Reference	1.74 (1.45–2.07)	2.39 (1.89–3.03)	2.09 (1.68–2.61)	2.28 (1.73–3.00)
Calendar year 2010+		Reference	1.89 (1.56–2.28)	2.17 (1.66–2.84)	2.31 (1.98–2.69)	2.86 (2.31–3.54)
One randomly sampled record per patient (any age and calendar time)		Reference	2.07 (1.78–2.42)	2.69 (2.22–3.26)	2.19 (1.87–2.56)	2.87 (2.28–3.61)
All	Propensity-matched (any or no coded indication)	Reference	1.57 (1.45–1.71)	1.88 (1.70–2.08)	1.82 (1.68–1.97)	2.06 (1.87–2.27)
	Propensity-matched (any or no coded indication and no inpatient/outpatient referral in 1 year before)	Reference	1.70 (1.39–2.06)	2.09 (1.60–2.73)	1.91 (1.67–2.18)	1.76 (1.50–2.06)
LRTI	Propensity-matched (LRTI)	Reference	1.57 (1.21–2.04)	1.95 (1.46–2.61)	1.48 (1.17–1.87)	2.36 (1.76–3.18)
URTI	Propensity-matched (URTI)	Reference	2.47 (2.08–2.93)	3.17 (2.60–3.86)	1.85 (1.55–2.21)	2.39 (1.87–3.05)
UTI	Propensity-matched (UTI)	Reference	1.12 (0.87–1.55)	1.28 (0.88–1.87)	1.57 (1.10–2.25)	1.65 (1.05–2.58)

Comparisons of the clinical codes between patients with lowest and highest histories of antibiotic use showed considerably higher recording of clinical codes indicating infections. The ten clinical codes with highest ORs were recurrent urinary tract infection (OR of 57.88), hidradenitis suppurativa (34.34), myringoplasty (31.55), chronic prostatitis (30.61), history of recurrent tonsillitis (29.67), frontal sinusitis (19.96), tonsillectomy (19.14), recurrent UTI (19.09), recurrent cystitis (16.88) and history of recurrent cystitis (16.49). The false discovery rate-adjusted *P* values were below 0.05 for all these clinical codes. Among the clinical codes with an OR > 1.5 in the quintile with highest history of antibiotic use compared to lowest quintile, there were no clinical codes describing conditions that can lead to immunosuppression.

## Discussion

Many patients in UK primary care repeatedly receive antibiotics. The level of prior antibiotic use was found to be a strong predictor of the pattern of changes in the risk of infection-related hospital admissions (mostly due to LRTI or pneumonia) after the antibiotic prescriptions. Patients with limited prior antibiotic use showed rapid and strong reductions in the risks, with risks returning to low levels within 2 months. In contrast, patients with higher levels of prior antibiotic use showed more moderate reductions in risk, with sustained higher risks over time. A strong dose-response was found between the patterns of changes in risk and level of prior antibiotic use.

There may be several potential explanations for the observed patterns of sustained higher risks of infection-related hospital admissions risks over time. The study findings could be related to confounding due to patients being immunocompromised and suffering more severe and more frequent infections, or colonization with resistant bacteria in patients previously admitted to the hospital. Another explanation could be a causal effect and involve gut microbiota. The intestinal commensal microbiota provides colonization resistance against pathogens. It may be possible that antibiotics cause dysbiosis (perturbations of the intestinal microbiota), contributing to the loss of colonization resistance followed by an increment of the resistome in the intestinal microbiota [17, 18]. A recent animal study reported that antibiotic use causes intestinal macrophages to become hyperresponsive to bacterial stimulation, possibly leading to increased susceptibility to infections [19]. Our study does not provide direct evidence to support any of these biological mechanisms. However, there is indirect evidence to support the mechanism of decreased antibiotic effectiveness with repeated use due to resistance. It was found that the severity of the infections (as measured in the first 3 days) was broadly comparable between the groups with different histories of antibiotic use (rather than increased in immunocompromised patients). Also, relatively higher risks were found in patients without comorbidity and younger patients (who are less likely to be immunocompromised); exclusion of patients previously referred to the hospital did no change results. The stratification by propensity score found that the patients most likely to have limited history of prior antibiotic use had the highest increases in the risks of infection-related hospital admissions. A review of the clinical codes in patients with more frequent histories of antibiotic use also did not show frequent presence of clinical conditions with immunosuppression. But whatever the aetiology of our findings of decreased antibiotic effectiveness with more frequent histories of use, there is no evidence from systematic reviews that frequent and repeated use of antibiotics is actually clinically effective. In the UK, guidelines for the treatment of common infections in primary care focus on the first episode of antibiotics and not repeated or frequent use. The only exception is the treatment guidelines for recurrent UTI although they do not cover the repeated use as observed in this study [20]. The lack of clinical evidence to support the frequent and repeated use of antibiotics should caution against this common practice. Antibiotic guardianship may have to consider reviews of patients who frequently and intermittently receive antibiotics.

There is some evidence in the literature that suggests a direct link between changes in microbiota and antibiotic exposure. A randomized trial in preschool children reported increased antibiotic resistance in the gut microbiota after a single course of antibiotics [21]. The possibility of microbiota dysbiosis due to antibiotic exposure is also supported indirectly by the review of Costelloe which found that individuals prescribed an antibiotic in primary care for a respiratory or urinary infection develop bacterial resistance to that antibiotic [6]. A recent large population-based study from Denmark found that antibiotic exposure before or during pregnancy was associated with increased risk of childhood infections leading to hospitalizations [22]. Further work is needed to test this hypothesis that changes in the gut microbiome and dysbiosis can lead to increases in infections with resistant bacteria.

This study has several strengths and limitations. A key strength was that two datasets were used including two very large populations of patients from both England and Wales and allowing replication of findings in independent datasets. The coding of clinical events may be suboptimal in UK primary care [23]; a large proportion of antibiotic prescriptions had no coded indication in the present study. But the primary outcome in this study was based on data independently collected in hospitals and more likely to be complete. Analyses including patients irrespective of a record of a common infection found comparable results. One key limitation of this study was that patients were not randomly allocated to different levels of prior antibiotic use. A randomized trial is impractical as patients cannot be randomized to different histories of use. There is the potential for residual confounding due to comorbidities of immune function. However, as outlined above, there were observed increases in risk of infection-related complications even in patients in the lower quintiles of prior antibiotic use. The differences between the various prior use groups were smaller shortly after the antibiotic prescriptions and larger in more distant time. We could not measure any antibiotics prescribed outside general practice (by e.g. dentists, walk-in centres, hospitals or emergency departments). This could confound the results if the propensity to prescribe antibiotics to particular patients would be opposite between GPs and other prescribers. Another limitation was that prophylactic antibiotic use was not captured. Prophylactic antibiotic use is recommended in patients with more severe COPD (this treatment guideline was introduced in 2010) [24]. However, patients with COPD had been excluded, and time-trend analyses showed effects of prior antibiotic use prior to 2010. Finally, a limitation was that antibiotic prescribing in hospitals and acute care was not captured in this study. However, the large majority of antibiotic prescribing occurs in primary care.

In conclusion, there is little evidence in the literature for the clinical effectiveness of repeated antibiotic use in primary care although is common practice. Repeated courses of antibiotics may have limited benefit and indicator of adverse outcomes. This is possibly due to dysbiosis of gut microbiota and resistance. Antibiotics should be used infrequently for common infections unless there is clear evidence of an infection with susceptible bacteria. Antimicrobial stewardship interventions should target these patients with high use of antibiotics but apparently limited value.

## Data Availability

The anonymized patient-level data used for this project cannot be shared for reasons of information governance. However, data can be obtained by application to CPRD and SAIL.
